# Better decision-making skills support tactical behaviour and reduce physical wear under physical fatigue in soccer

**DOI:** 10.3389/fphys.2023.1116924

**Published:** 2023-04-12

**Authors:** Felipe Dambroz, Israel Teoldo

**Affiliations:** Department of Physical Education, Centre of Research and Studies in Soccer (NUPEF), Universidade Federal de Viçosa, Viçosa, Brazil

**Keywords:** fatigue, assessment, pacing, tactics, football

## Abstract

The purpose of this study was to verify whether decision-making skills influence the tactical behavior and physical performance of soccer players under acute physical fatigue, assessed in an actual game-play. The sample was comprised of 24 trained soccer players (18.25 ± 1.48 years old) from two Brazilian clubs grouped into two categories with 12 players each (with high and low decision-making skills). The assessment of decision making, tactical behavior, and physical performance were carried out using TacticUP^®^, FUT-SAT, and GPSports^®^, respectively. Acute physical fatigue was induced through the T-SAFT90 test. Results showed that under acute physical fatigue players with high decision-making skills maintained tactical behavior efficiency and had their paces reduced, in addition to displaying reduced total distance covered (*p* < 0.001), number of accelerations (*p* < 0.001), and decelerations (*p* < 0.001), and average movement speed (*p* < 0.001). On the other hand, players with low decision-making skills displayed reduced tactical behavior efficiency (*p* = 0.002) and maintained their movement pace under physical fatigue. It is concluded that decision-making skills contribute to players’ tactical behavior efficiency under acute physical fatigue, besides promoting reduced physical strain in movement actions throughout the field.

## 1 Introduction

Over the last decade, decision-making has drawn attention and received growing research interest with the purpose of understanding the processes and mechanisms that sustain this skill in different game contexts (Teoldo et al., 2022). In general, decision-making skills are related to the player’s capacity to integrate existing information in the environment with the knowledge and experience acquired throughout the years to select the appropriate action that needs to be performed (Baker et al., 2003). Therefore, in soccer, the development of decision-making skills allows the players to deal in an efficient fashion with the events that randomly emerge during the game (Roca and Williams, 2017).

Research has indicated that players who are efficient in performing tactical actions display higher decision-making skills (Assis et al., 2020; Gonçalves et al., 2020). Besides, studies show that players who make better decisions display superior game-reading skills and are more efficient in identifying relevant environmental information (Den Hartigh et al., 2018; Machado and Teoldo, 2020). During the game, these characteristics enable players to be more efficient when moving across the playing field (Gonzaga et al., 2014; Cardoso et al., 2019). Thus, well-developed decision-making skills may be particularly relevant at the end of matches, when the ability to perform movement actions is reduced due to the player’s state of physical fatigue (Iodice et al., 2017; Coutinho et al., 2018).

Acute physical fatigue is described as a reduction in maximal voluntary muscle strength and inability to maintain a certain intensity or power level during exercises (Gandevia, 2001). Consequently, acute physical fatigue has been regarded as one of the main aspects that impact players’ performance (Carling et al., 2018). Previous studies with soccer players, based on the model proposed by Edwards and Noakes (2009), suggest that in situations of physical strain, players tend to consciously and/or subconsciously adapt their movement pace as a strategy to maintain performance (Nassis et al., 2015; Brito et al., 2016). For instance, in the study by Coutinho and colleagues (2018), the authors verified that players reduce the movement pace and adopt more stable tactical behaviors under conditions of muscle fatigue. However, this study did not consider the level of decision-making skills of the group and only assessed the collective tactical behavior of the team. Therefore, in order to move forward on this topic, it is essential to consider players’ individual tactical behavior efficiency, i.e., the quality of tactical actions performed under the state of physical fatigue.

Considering the above, although decision-making has not been explored as a characteristic for group categorization, this skill may mediate an aspect of players’ performances under the state of physical fatigue. Studies on this topic indicate that players with higher levels of decision-making skills display better performance indexes (Roca et al., 2011; Murr et al., 2021). Also, in laboratory settings, elite players experienced less perceptual and cognitive changes and adapted their patterns of information processing so as to maintain performance when compared to their lower-level counterparts in situation of fatigue (Casanova et al., 2013; Skala and Zemková, 2022). Nevertheless, scientific literature still lacks studies with players on the same competitive level under the state of acute physical fatigue categorized by their decision-making skills, and subsequently assessed through a field test. This is particularly relevant considering that players’ tactical behavior and performance are intimately related to their decision-making skills (Assis et al., 2020), and emerge from the interaction of the tactical, physical, technical and cognitive components of the game (Teoldo et al., 2022). Hence, in order to improve the assessment and training processes, as well as to identify performance parameters that enable future interventions, the purpose of the present study was to verify whether decision-making skills influence the tactical behavior and physical performance of soccer players under the state of acute physical fatigue assessed during game-play.

## 2 Materials and methods

### 2.1 Sample

The sample was comprised of 24 trained male soccer players (mean age: 18.3 ± 1.5 years) from two Brazilian clubs (McKay et al., 2022). As inclusion criteria, players should be participating in systematic training sessions with duration of 90 min, at least three times a week, and play in tournaments at regional and state levels.

### 2.2 Ethical procedures

The participants were previously informed about the objective of the study. The present study was approved by the Ethics Committee in Research with Human Beings (no. 3.208.190) and conformed to the norms of the National Health Council (CNS 466/2012) and the Declaration of Helsinki (2013). To take part in the study, the participants aged 18 years and older signed an informed consent form; the participants who were minors signed an assent form, while the participants’ legal guardians signed an informed consent form.

### 2.3 Instruments and procedures

#### 2.3.1 Experimental design

**FIGURE 1:**
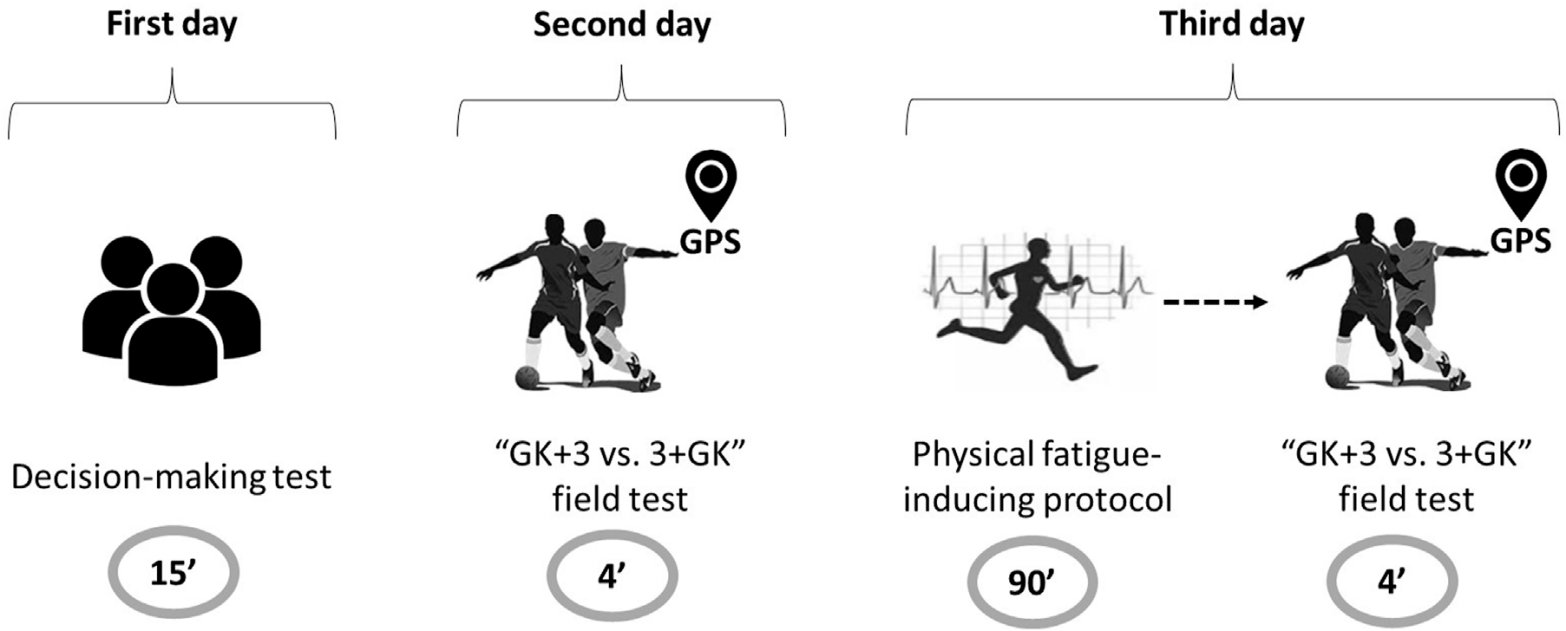
The representation of experimental design.

Participants were assessed on tree different days (Figure 1).

In the first day, participants took the decision-making test (TacticUP^®^). In the second day, the “control” condition was applied, in which participants performed the “GK+3 vs. 3+ GK” field test, monitored by GPS units. Teams were composed by one defender, one midfielder and one forward, and were assembled according to players’ technical levels following the head coach’s criteria in order to balance the level of the games (Casamichana and Castellano, 2010). In the third day, following a 48-h interval between assessments, participants were assessed in the “physical fatigue” condition. Participants performed the T-SAFT^90^ and, subsequently, the “GK+3 vs. 3+ GK” tests. The interval between tests was of approximately 1 min, which was the time necessary to move between test locations.

Participants were advised not to undergo any kind of physical exercise and to avoid the consumption of any caffeinated or alcoholic drinks up to 48 h prior to the interventions, as well as to sleep between 6 and 8 h in the previous night. In order to ensure that all participants consumed the same food, a pre-test standardized meal was provided (1 hour before the start of the tests), so as to meet the estimated required 18% of energy to each participant (approximately 380 kcal, 68 g CHO, 11 g PRO and 7 g Fat). Water intake during the experiment was *ad libitum*. The average room temperature and humidity on the day the participants were submitted to the physical fatigue protocol was of 21°C ± 1°C and 57% ± 1%, respectively.

#### 2.3.2 Physical and physiological measures

This study employed the CR10 Scale, proposed by Foster et al. (1995), whose reference is a scale that varies from 0 (rested) to 10 (maximum effort) to indicate the rating of perceived effort (RPE) of an activity. The RPE scale is used to assess the individual’s perception of effort during exercise and the workload of the activity in which participants answered to the following question: “How was your session?“. Thirty minutes after the end of the control and physical fatigue protocols participants informed the main researchers about the RPE value that corresponded to the effort perceived during sessions (Impellizzeri et al., 2004).

#### 2.3.3 Decision making

Decision-making data was collected using the TacticUP^®^ video test for soccer, an online assessment platform (www.tacticup.com.br) based on the core tactical principles of soccer (Teoldo et al., 2022). The TacticUP^®^ includes a sequence of 11 vs. 11 videos, which enable the assessment of the individual’s ability to make decisions in both phases of play (offensive and defensive), in actions with and without the ball, that take place near and far from the ball. Participants performed 36 trials in random order with three scenes for each tactical principle, besides having three scenes for familiarizing with the test. In each scene, four response options are displayed and participants are asked to select the most appropriate course of action for every scene. At the end, the test score is given. In the present study, the TacticUP^®^ measure that described players’ decision-making skills was the overall score, i.e., the mean of the performance indexes of the offensive and defensive phases (for details, see Machado and Teoldo, 2020).

All procedures were clarified to the participants prior to the start of the tasks, and the researcher was always available to answer potential questions. The TacticUP^®^ video test was presented individually for each participant on a 15-inch screen laptop computer LENOVO model 330 (Intel CoreTM i5 processor). The test procedure was carried out in a closed environment (indoors) and without external interference. The entire test procedure lasted around 15 min for each participant.

#### 2.3.4 Tactical behaviour

**FIGURE 2:**
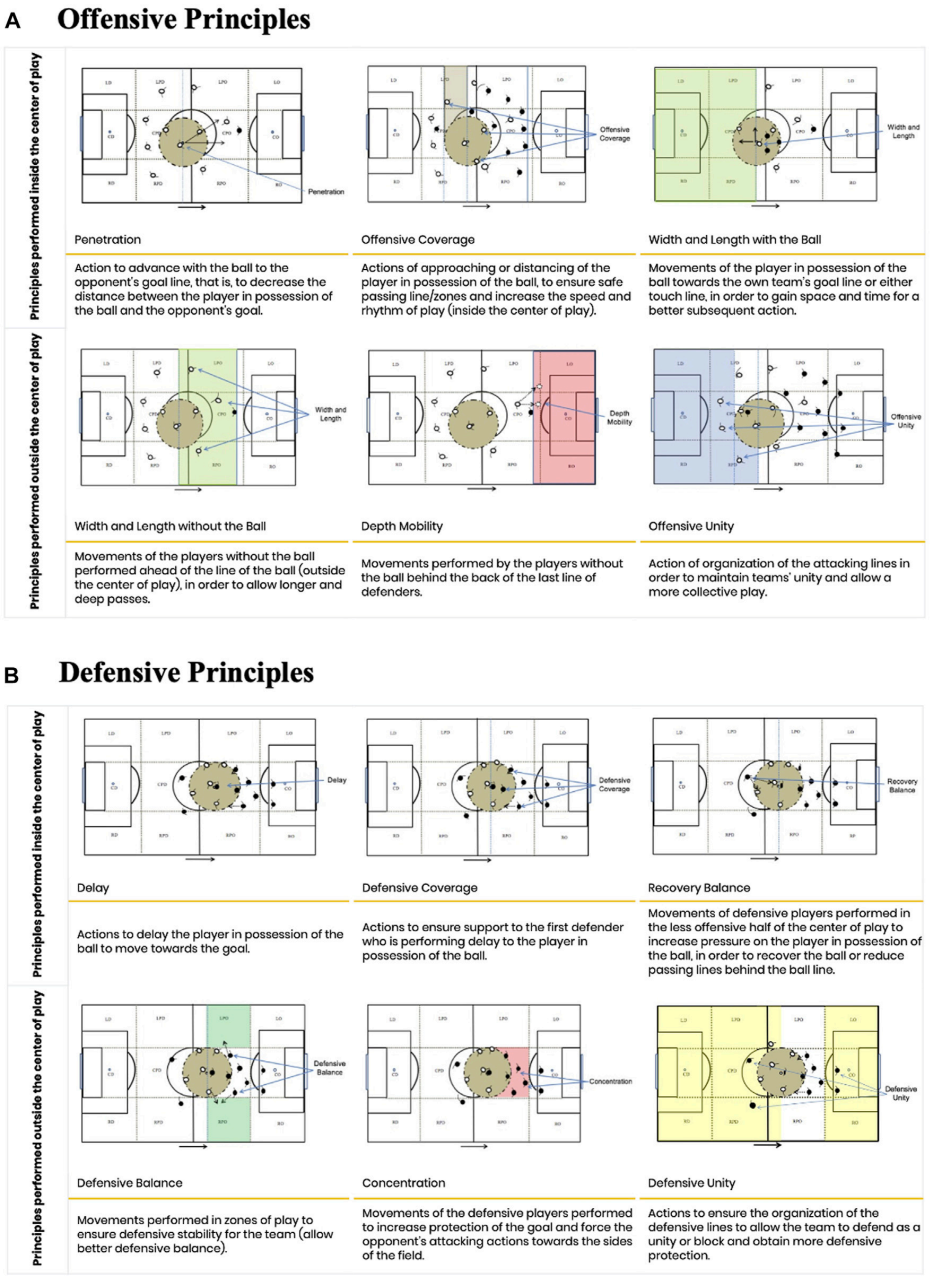
Description of the core tactical principles of soccer **(A)** offensive and **(B)** defensive.

The System of Tactical Assessment in Soccer (FUT-SAT) was used to assess participants’ tactical behaviour (Costa et al., 2011). Tactical assessment performed through FUT-SAT is based on the ten core tactical principles of soccer; five offensive and five defensive principles with and without the ball that, performed either inside or outside the centre of play (Figure 2). The centre of play is a dynamic spatial reference, characterized by a circumference of 9.15 m radius from the location of the ball, in which movements and decisions during the game occur at greater pace (Teoldo et al., 2022). FUT-SAT was performed in a small-sided game (GK+3 vs. 3+ GK), in a 36-m length and 27-m wide pitch, with duration of 4 min and 30 s of familiarization, and according to the official rules of soccer.

The FUT-SAT protocol comprises three procedures: the first consists of analyzing the players’ actions during a game, in which ball possession is considered a unit to distinguish between defensive and offensive phases; the second procedure refers to the assessment, classification, and recording of tactical actions based on spatial references of the field; and the third and last procedure refers to the calculation of variables within the “Tactical Actions” and “Percentage of Correct Actions” categories. In the present study, the percentage of accuracy and the number of tactical actions were the FUT-SAT variables used to describe the efficiency and frequency of the tactical actions, respectively. Following data collection, data analysis was performed according to the procedures proposed by Costa and colleagues (2011).

#### 2.3.5 Physical performance

During the field and the T-SAFT90 tests, GPS units (SPI-HPU—GPSports^®^, Canberra, Australia) were used to obtain data on players’ physical performance, with a sampling rate of 5 Hz for tracking players’ positions in the field. The categorization of the intensity zones of distance covered was based on Bradley et al. (2009). The values of distance covered were grouped as follow: standing (<0.7 km/h^-1^), walking (0.8–7.1 km/h^-1^), jogging (7.2–14.3 km/h^-1^), running (14.4–19.7 km/h^-1^), high-speed running (19.8–25.1 km/h^-1^), and sprinting (>25.1 km/h^-1^). In addition, the following parameters were also considered: total distance covered (m), average speed (km/h), number of sprints (AU), number of accelerations (AU) and number of decelerations (AU). The GPSports^®^ built-in software was used to analyze physical performance.

#### 2.3.6 Physical fatigue-inducing protocol

The T-SAFT90 test was used as a physical fatigue-inducing protocol. The T-SAFT90 is a protocol that simulates a soccer game with duration of 90 min, which reflects the metabolic demands of the game with the purpose of prompting the same responses regarding the internal and external loads. This task was used based on its potential to induce actions of muscle damage, such as accelerations, decelerations, and changes of directions. The T-SAFT90 incorporates activities with the ball, including running, dribbling, shooting, passing and jumps, in an intermittent fashion and varying speeds, such as in an actual soccer match.

The fatigue protocol consisted of 2 × 45 min of exercise with 15 min passive rest period during halftime. Both halves were divided in six blocks. These blocks consisted of 15 min of exercise. During the T-SAFT90, players covered 11,1 km divided in five speed zone: 0 km standing (0 km.h-1); 3.48 km walking (∼5.2 km h^-1^); 5.58 km jogging (∼10.5 km h^-1^); 1.68 km striding (∼15 km h^-1^) and 0.36 km sprinting (maximal effort). In addition to these, they performed ball dribbling 0.36 km, 24 short passes and 12 shoots on target. In relation to the acceleration and deceleration, the players performed 1,269 changes in speed (every 4.3 s), 888 changes in direction (180°), 444 cutting maneuvers and 12 jumps (Silva and Lovell, 2020). In previous studies, the type and intensity of these actions resulted in increased blood markers, such as creatine kinase, cortisol, leukocytes, neutrophils, myoglobin, neutrophils, lymphocytes, as well as increased muscle pain and perceived effort (Silva et al., 2018).

### 2.4 Statistical analysis

Descriptive analysis (means and standard deviation) was used to describe the sample characteristics. Distribution normality for all variables was verified using the Shapiro-Wilk test. The sample was categorized in two groups according to the score in the “general” item (average performance index in the offensive and defensive phases) of the TacticUP^®^ video test. In order to classify decision-making skills, the group with higher value (≥50th percentile) was named “High Skills Group” (n = 12), whereas the group with lower value (<50th percentile) was named “Low Skills Group” (n = 12). The values obtained for the groups were compared using the *t*-test for independent samples.

The paired *t*-test and Wilcoxon test were used to compare tactical behavior and physical performance between the “control” and “physical fatigue” conditions among groups. The test-retest method was used to calculate reliability, and the values of Cohen’s Kappa were used. Intra-observer reliability was carried out following a 3-week interval, so as to avoid task familiarity issues (Robinson and O’Donoghue, 2007). Out of 3,498 tactical actions, 385 actions were reassessed, representing 11% of the sample, which is higher than the reference value (10%) proposed by literature (Tabachnick and Fidell, 2007). Two observers participated in this procedure. Reliability values ranged between 84% and 95% for the intra-observer reliability, and between 83% and 98% for the inter-observer reliability. Both ranges are within the interval classified as “almost perfect” (0.81–1), which ensures a high level of agreement between observers (Landis and Koch, 1977). The magnitudes of the effect sizes were classified as: null (<0.20), small (0.21–0.60), medium (0.61–1.20), and large (>1.20) (Hopkins et al., 2009).

The significance level was set at *p* < 0.05. All statistical procedures were performed using IBM SPSS (*Statistical Package for Social Sciences*) for Windows^®^, version 24.0.

## 3 Results

### 3.1 Physical and physiological measures

Participants displayed higher perceived effort under the physical fatigue condition (7.9 ± 1.1 AU) when compared to the control condition (1.1 ± 1.1 AU) (z = −6.06 *p* < 0.001; large effect size), which indicates that, on average, players considered the physical fatigue condition “very hard”.

### 3.2 Decision making

The mean values of decision making for the “High Skills” and “Low Skills” groups were significantly different (t_(11)_ = −7.19; *p* < 0.001; *d* = 2.93) (Table 1).

**TABLE 1:** Comparison of decision-making values between the high skills and low skills groups.

High skills group (n = 12)	Low skills group (n = 12)	CI 95%		
*M DP*	*M DP*	*Inf.—Sup*	*p*	*d*
72.57 ± 3.95	61.37 ± 3.68	−14.44—−7.97	<0.001^a^	2.93

^a^ Significant difference at *p* < 0.05.

### 3.3 Tactical bahaviour and physical performance

In the group with higher decision-making skills, no significant differences were found regarding tactical behavior efficiency for the comparison between the “control” and “physical fatigue” conditions (Table 2). Conversely, significant differences were found for physical performance in the comparison between both conditions, as players displayed decreased distance covered in jogging (*t*_*(11)*_ = 6.40; *p* < 0.001; *d* = 1.73), running (*t*_*(11)*_ = 3.89; *p* = 0.003; *d* = 1.71), high-speed running (*t*_*(11)*_ = 4.32; *p* = 0.001; *d* = 1.33), average speed (*t*_*(11)*_ = 8.20; *p* < 0.001; *d* = 2.82), number of sprints (*z* = −2.40; *p* = 0.016; *d* = 1.11), accelerations (*t*_*(11)*_ = 5.13; *p* < 0.001; *d* = 1.27), and decelerations (*t*_*(11)*_ = 5.46; *p* < 0.001; *d* = 1.40) under the “physical fatigue” condition (Table 2).

**TABLE 2:** Comparison of the tactical behavior and physical performance between the control and physical fatigue conditions of high and low skills decision-making groups.

Variable	High skills group (n = 12)					Low skills group (n = 12)				
	Control	Physical fatigue		95% CI		Control	Physical fatigue		95% CI	
	*M SD*	*M SD*	*p*	*Lower—Upper*	*d*	M SD	*M SD*	*p*	*Lower— Upper*	*d*
Tactical Behavior Efficiency										
Offensive	86.36 ± 12.30	87.63 ± 7.61	0.657	−7.35—4.83	0.12	83.93 ± 9.09	79.2 ± 8.56	0.095	−11.18—0.74	0.54
Defensive	79.02 ± 13.21	71.69 ± 10.24	0.112	−2.01—16.68	0.54	73.28 ± 13.92	57.35 ± 10.2	0.002^a^	7.13—24.74	1.31
Total	82.6 ± 12.23	78.78 ± 7.06	0.248	−3.07—10.72	0.38	78.79 ± 10.67	69.54 ± 6.08	0.002^a^	4.38—14.12	1.07
Physical Performance										
Total distance covered (m)	498.02 ± 31.18	398.53 ± 42.19	<0.001^a^	73.21—125.76	2.68	505.63 ± 38.66	473.83 ± 40.20	0.071	−3.18—66.78	0.81
Standing (m)	1.61 ± 0.62	1.69 ± 0.75	0.784	−6.15—27,18	0.11	1.53 ± 0.72	1.50 ± 0.53	0.876	−0.40—0.47	0.05
Walking (m)	177.43 ± 20.40	185.51 ± 19.05	0.308	−0.72—0.56	0.41	179.72 ± 12.05	180.81 ± 18.36	0.875	−15.86—13.69	0.07
Jogging (m)	233.08 ± 34.51	164.11 ± 44.75	<0.001^a^	45.24—92.69	1.73	238.30 ± 22.63	225 66 ± 41.62	0.320	−14.09—39.36	0.38
Running (m)	68.72 ± 15.76	42.08 ± 15.42	0.003^a^	11.58—41.70	1.71	75.05 ± 24.53	53.40 ± 11.88	0.033^a^	2.13—41.17	1.12
High-speed running (m)	16.71 ± 11.26	5.15 ± 4.93	0.001^a^	5.67—17.46	1.33	10.68 ± 11.29	12.46 ± 12.96	0.754	−13.96—10.40	0.15
Sprinting (m)	0.49 ± 1.67	0.48 ± 1.67	0.317	0.00—0.00	0.01	0.35 ± 0.86	0.26 ± 0.47	0.465	−0.69—0.50	0.13
Average speed (km/h)	7.33 ± 0.46	5.82 ± 0.60	<0.001^a^	1.11—1.93	2.82	7.44 ± 0.57	6.98 ± 0.60	0.084	0.07—1.00	0.79
Sprints (AU)	1.42 ± 1.17	0.42 ± 0.51	0.016^a^	−1.50—0.00	1.11	0.83 ± 1.03	1.08 ± 1.08	0.587	−1.00—1.00	0.24
N. of Accel. (AU.)	47.25 ± 5.43	39.33 ± 6.99	<0.001^a^	4.52—11.32	1.27	47.08 ± 5.40	45.75 ± 6.02	0.572	−3.70—6.37	0.23
N. of Decel. (AU)	34.17 ± 4.53	26.58 ± 6.16	<0.001^a^	4.53—10.64	1.40	33.42 ± 4.62	33.50 ± 4.58	0.969	−5.00—3.50	0.02

^a^ Significant difference at *p* < 0.05; a. u. arbitrary unit.

As for the group with lower decision-making skills, defensive (*t*_*(11)*_ = 3.98; *p* = 0.002; *d* = 1.31) and overall tactical behavior efficiency (*t*_*(11)*_ = 4.18; *p* = 0.002; *d* = 1.07) were found lower under the physical fatigue condition (Table 3). On the other hand, with respect to physical performance, participants displayed a decrease only for the distances covered in running (*t*_*(11)*_ = 2.44; *p* = 0.033; *d* = 1.12) under the state of physical fatigue (Table 2).

## 4 Discussion

The purpose of the present study was to verify whether decision-making skills influence the tactical behavior and physical performance of soccer players submitted to acute physical fatigue and assessed during the game. Results showed that players with higher decision-making skills maintained tactical behavior efficiency and decreased their pace under the state of acute physical fatigue. On the other hand, players with lower decision-making skills displayed reduced tactical behavior efficiency while maintaining their pace under the state of acute physical fatigue.

With respect to the findings on tactical behavior efficiency, players with higher decision-making skills maintained the quality of tactical actions under acute physical fatigue. Decision-making skills refer to players’ ability to quickly extract and process visual information from the game, as well as to retrieve scenarios that have already been experienced through working memory (Roca et al., 2018; Cardoso et al., 2021). Hence, even under physical fatigue this ability does not seem to vary, which consequently would allow players to maintain their game reading skills and efficiently perform tactical actions (Den Hartigh et al., 2018; Assis et al., 2020). On the other hand, players with lower decision-making skills displayed reduced efficiency when performing tactical actions under acute physical fatigue. This reduction may have a direct and negative impact on individual and collective performances, especially in the final moments of a match. Previous empirical studies showed that at the end of matches players commit a higher number of passing errors (Barte et al., 2019), and teams concede more goals (Kubayi and Toriola, 2019) as a result of accumulated physical fatigue. Although the studies did not account for players’ decision making, this skill seems to be relevant to enable players to overcome the limitations posed by physical strain.

Another important finding is that players with higher decision-making skills maintained tactical behavior efficiency under acute physical fatigue by making less physical effort. Conversely, players with lower decision-making skills, in addition to being less efficient when performing tactical actions—i.e., committing more mistakes when moving and positioning themselves on the field –, displayed similar physical effort compared to when they were rested, thus showing greater physical strain than players with higher decision-making skills. In this scenario, it is possible to understand the maintenance of physical performance under acute physical fatigue as a result of difficulties in managing playing space, or even as a physical compensation for the decreased tactical behavior efficiency. This behavior may lead to higher injury risk, since players under physical fatigue display impaired movement mechanics and inability to maintain contraction force (Gualtieri et al., 2020; Raya-González et al., 2022). In general, previous studies showed that teams under physical strain display a more stable collective tactical behavior, with the purpose to reduce the need to move at high speeds (Sampaio et al., 2014; Coutinho et al., 2018). However, participants in the aforementioned studies were not categorized according to their decision-making skills, and the measures used referrer to collective tactical behavior. Consequently, the present study was the first to consider measures of tactical actions and values of players’ individual movements, besides showing that players’ responses vary according to their decision-making skills.

Based on the findings of the present study and aiming to foster sports success, it is recommended that coaches try to develop players’ decision-making. Accordingly, accumulated experience in the sport and access to quality training throughout the development process have been deemed essential for improving decision-making (Musculus et al., 2018; O’Connor et al., 2018). For instance, the study by Machado et al. (2020) showed that the higher number of accumulated hours in activities related to collective tactical development with elements that demand great decision making (e.g., small-sided and conditioned games) were the ones that contributed the most to the acquisition of decision-making skills by the players throughout their sports development. These activities were all characterized by simulating environments that simultaneously demand perceptual, cognitive, and motor skills from players, in addition to including aspects such as great decision-making and other game-related elements. Therefore, it is possible that, by developing their decision-making skills, players are capable of maintaining performance under fatigue, since the ability acquired throughout the development process allows them to integrate information existing in the working memory, thus facilitating storage and retrieval of game-specific information (Ericsson and Kintsch, 1995). In this scenario, it seems plausible to suggest that this ability enables players to find solutions for the problems the game systematically poses, despite their motor limitations.

In terms of practical implications, soccer players tend to constantly face situations of physical wear during training sessions, matches and throughout the competitive season (Bengtsson et al., 2018). Thus, as players with higher decision-making skills are able to maintain the quality of their actions and display reduced physical strain when tired, developing players’ decision-making may be an aspect that helps maintaining performance and even reduces the risk of injuries during matches and seasons. Hence, it is necessary to objectively assess players’ decision-making and tactical behavior so as to provide coaches with parameters regarding players’ development (Roca and Ford, 2020). These findings may provide a solid foundation for coaches to meet the demands of his/her group of players, by regulating the appropriate use of pedagogical principles with the purpose of stimulating the development of decision making (O’Connor et al., 2018; Roca and Ford, 2020).

## 5 Conclusion

In sum, players with higher decision-making skills maintain tactical behavior efficiency and decreased their physical strain when assessed during the game under the state of acute physical fatigue. Conversely, players with lower decision-making skills displayed reduced tactical behavior efficiency and higher physical strain. Therefore, the findings of this study indicate that decision-making skills are a conditioning factor for players’ movement and positioning under the state of acute physical fatigue, thus influencing individual and collective performances.

## 6 Limitations

Although the experimental design of the present study is innovative, the results must be analyzed with caution due to the small sample size, which makes possible the occurrence of type-II errors. In addition, the playing time participants underwent was short. Future research on the influence of acute physical fatigue on performance should control for players’ decision-making skills since we identified them as a variable that plays a role in players’ tactical efficiency and physical performance under acute physical fatigue. This kind of approach aims to improve the assessment process and to allow the transfer and applicability of findings to training. Additionally, since this is the first study that investigates decision-making as a mediating aspect of players’ tactical and physical performance under acute physical fatigue, it is recommended that future studies use larger samples with different ages, genders, and competitive levels.

## 7 Practical applications

1) Players with higher decision-making skills under acute physical fatigue are able to maintain the quality of their actions and display reduced physical strain when tired.
2) Players with lower decision-making skills under acute physical fatigue committing more mistakes when moving and positioning themselves on the field and displayed similar physical effort compared to when they were rested.
3) Players with lower decision-making skills compensate the decrease of tactical behavior efficiency maintenance of physical performance.
4) Decision making ability well establish enables players to find solutions for the problems the game systematically poses, despite their motor limitations.
5) Developing players’ decision-making is an aspect that helps maintaining performance and even on the reduction of injury risk during matches and seasons

## Data Availability

The raw data supporting the conclusion of this article will be made available by the authors, without undue reservation.
